# Cannabidiol (CBD) as a novel treatment in the early phases of psychosis

**DOI:** 10.1007/s00213-021-05905-9

**Published:** 2021-07-13

**Authors:** Edward Chesney, Dominic Oliver, Philip McGuire

**Affiliations:** 1 Department of Psychosis Studies, Institute of Psychiatry, Psychology & Neuroscience, King’s College London, London, UK; 2 South London and Maudsley NHS Foundation Trust, London, UK; 3 National Institute for Health Research Maudsley Biomedical Research Centre, London, UK

**Keywords:** Cannabidiol, CBD, Psychosis, Schizophrenia, First-episode psychosis, Clinical high risk, Cannabinoids, Antipsychotics, Treatment

## Abstract

The pharmacological interventions available for individuals in the early stages of psychosis are extremely limited. For those at clinical high risk for psychosis, there is no licensed treatment available. For those with first-episode psychosis, all licensed antipsychotic medications act via dopamine D_2_ receptors. While treatment with antipsychotics is transformative in some patients, in others, it is ineffective. In addition, these medications can often cause adverse effects which make patients reluctant to take them. This is a particular problem in the early phases of psychosis, when patients are being treated for the first time, as unpleasant experiences may colour their future attitude towards treatment. Recent research has suggested that cannabidiol (CBD), a compound found in the *Cannabis sativa* plant, may have antipsychotic effects and relatively few adverse effects and could therefore be an ideal treatment for the early phases of psychosis, when minimising adverse effects is a clinical priority. In this review, we consider CBD’s potential as a treatment in the clinical high risk and first-episode stages of psychosis. First, we describe the limitations of existing treatments at these two stages. We then describe what is known of CBD’s mechanisms of action, effectiveness as a treatment for psychosis, adverse effects and acceptability to patients. We discuss how some of the outstanding issues about the utility of CBD in the early phases of psychosis may be resolved through ongoing clinical trials. Finally, we consider the impact of recreational cannabis use and over-the-counter cannabinoids preparations and discuss the potential therapeutic role of other compounds that modulate the endocannabinoid system in psychosis.

## Introduction

Psychosis is mainly treated using antipsychotic drugs, which were first introduced in the 1950s. These medicines are still the only effective pharmacological treatment for the disorder. However, in around a third of patients, they do not relieve psychotic symptoms (Boter et al. 2009), and even when they are effective, they can cause serious side effects which often make patients reluctant to take them (Sendt et al. 2015). This is a particular problem in the early phases of psychosis, when people are being treated for the first time, as unpleasant experiences at this stage may colour their attitude towards treatment in the longer term (Liu and Demjaha 2013; Bjornestad et al. 2017). There is thus a long-standing need for alternative pharmacological treatments.

Cannabidiol (CBD) is a naturally occurring phytocannabinoid produced by the *Cannabis sativa* plant. It is an approved treatment for rare childhood epilepsy syndromes (Wise 2018) and, unlike delta-9-tetrahydrocannabinol (THC), is not intoxicating (Schoedel et al. 2018). The first evidence that CBD may have beneficial effects in patients with psychosis came from case studies (Zuardi et al. 1995, 2006, 2009; Makiol and Kluge 2019). More recently, small-scale clinical trials have compared the effects of CBD with those of antipsychotic medication or placebo, with the majority reporting positive results and all reporting minimal adverse effects (Leweke et al. 2012; Boggs et al. 2018; McGuire et al. 2018) (see below). In the present review, we examine the evidence that CBD may be useful as a novel treatment in psychosis, with a particular emphasis on its use in the early phases of the disorder.

## Methods

A critical review of the past literature was conducted. Relevant articles were retrieved through targeted searches on international databases (Google Scholar and Clinicaltrials.gov) using the terms ‘cannabidiol’, ‘CBD’, ‘schizophrenia’ and ‘psychosis’ and critically reviewed by the authors of the paper. We additionally reviewed the citations of relevant systematic reviews and consulted with experts in the fields of cannabinoid psychopharmacology and psychosis. The review did not follow a systematic literature search, data extraction or reporting approach.

## Review

### Limitations of existing interventions for the clinical high risk state

The majority of patients with psychosis can recall experiencing an earlier prodromal phase (Jackson et al. 1995), typically featuring a decline in overall functioning and the emergence of ‘attenuated’ psychotic symptoms (Fusar-Poli et al. 2020). Anxiety and depressive symptoms are also common. Prospective studies indicate that 15–30% of people presenting with this syndrome will go on to develop a first episode of psychosis within 3 years (Fusar-Poli et al. 2020), which is why it is referred to as a clinical high risk (CHR) state.

In the short term, clinical services for people at CHR aim to relieve the presenting symptoms and address any related psychological or social problems. In the longer term, they aim to reduce the risk of progression to a psychotic disorder. The interventions offered vary between centres and usually include case management and clinical monitoring (Schmidt et al. 2015). In addition, a number of specific pharmacological and psychological interventions have been evaluated in clinical trials over the last 20 years. However, the results have been inconsistent, and recent meta-analyses indicate that neither antipsychotic medications, cognitive behavioural therapy, family therapy nor omega-3 fatty acids are more effective than case management alone in reducing severity of attenuated positive psychotic symptoms or the risk of developing a psychotic disorder (Davies et al. 2018a, b). Furthermore, as they are antipsychotic naive and often younger, CHR individuals are more sensitive to the adverse effects of antipsychotics than patients with established psychosis (Liu and Demjaha 2013; Stafford et al. 2015) and few are willing to take them (Welsh and Tiffin 2014). Thus, at present, there is no licensed treatment for either the presenting symptoms of the CHR state or for reducing the risk of later psychosis in this group. This represents a major unmet clinical need.

### Limitations of existing treatments for first-episode psychosis

Antipsychotic medication is the most important component in the treatment of first-episode psychosis. In the short term, it is used to control the acute presenting symptoms (Huhn et al. 2019). Once these have remitted, continuing with treatment reduces the risk of subsequent relapse (Kishi et al. 2019). In about two-thirds of first-episode patients, the response to treatment of their presenting symptoms is good (Boter et al. 2009) and requires relatively low doses of antipsychotic medication. However, in about a third, these drugs are less effective. In addition, the beneficial effects of these drugs are mainly limited to positive psychotic symptoms; they have less impact on cognitive impairments or negative symptoms (Keefe et al. 2007; Krause et al. 2018). A further issue is that patients with psychosis are often reluctant to take antipsychotic medications, because of their reputation for side effects (Sendt et al. 2015) and because they are associated with schizophrenia, which is perceived as stigmatising (Yılmaz and Okanlı 2015). This reluctance is particularly evident in first-episode patients after their initial symptoms have resolved, as the benefits of prophylactic treatment may not be clear until after a relapse has occurred.

There is thus a clear need for alternative treatments. At present, if initial treatment is ineffective, standard clinical practice involves switching to a different antipsychotic medication. However, there is surprisingly little evidence that this is effective (Kahn et al. 2018). The only treatment that is effective when conventional antipsychotic medication has failed is with clozapine, an antipsychotic with a unique pharmacological profile (Siskind et al. 2016). However, clozapine can only be prescribed when at least two different antipsychotics have been ineffective, so it is not available to patients at the onset of psychosis or after their first antipsychotic treatment. Moreover, clinicians are sometimes reluctant to initiate treatment with clozapine due to the risk of serious adverse effects and the need for regular blood monitoring (Howes et al. 2012).

### The mechanism of action of CBD in psychosis

CBD is a particularly interesting candidate as a novel treatment for psychosis because its molecular mechanism of action appears to be different to that of antipsychotic medications, which are either antagonists or partial agonists at the dopamine D_2_ receptor (Kaar et al. 2020). CBD has a variety of central actions that could plausibly contribute to an effect on psychosis. One putative mechanism is direct activity at cannabinoid (CB) receptors. Both CB_1_ and CB_2_ receptors have been proposed as relevant targets (Zhang et al. 2014; Cortez et al. 2020; Borgan et al. 2021). CBD is a negative allosteric modulator of the receptors, limiting their response to their endogenous ligands: the endocannabinoids (Laprairie et al. 2015; Martínez-Pinilla et al. 2017). At much higher concentrations, CBD may act as an antagonist at the orthosteric sites of CB_1_ and CB_2_ receptors, although it seems unlikely that this occurs at clinically relevant concentrations (McPartland et al. 2015). It has also been proposed that CBD can prevent the internalisation of CB_1_ receptors (Laprairie et al. 2015) and could therefore help normalise the abnormally low CB_1_ receptor densities observed in patient populations (Borgan et al. 2021). However, the effect of CBD on this measure has not yet been investigated in vivo.

CBD may also act by inhibiting the metabolism of endocannabinoids. In the first clinical trial in psychosis, CBD treatment was associated with increased anandamide levels which were in turn correlated with reductions in psychotic symptoms (Leweke et al. 2012). In support of this theory, a recent study found that the levels of fatty acid amide hydrolase (FAAH), the enzyme which metabolises anandamide and other related ligands, were inversely correlated with severity of psychotic symptoms in patients with psychosis (Watts et al. 2020). It has been suggested that the exact mechanism of action may be via interruption of the fatty acid-binding proteins which transport endocannabinoids intracellularly (Elmes et al. 2015). Other ligands, such as palmitoylethanolamine, and effects of CBD on other receptors, such as GPR55 and TRPV1 (which are related to the endocannabinoid system), could also have a role, but the evidence supporting these is relatively sparse and is still limited to data from pre-clinical studies (Tzavara et al. 2006; Ryberg et al. 2007; Long et al. 2009; Muller et al. 2020).

Serotonergic receptors have also been proposed as a relevant target. Animal research using the NMDA receptor antagonist MK-801, a pharmacological model of psychosis, found that CBD’s antipsychotic effect can be blocked by a 5-HT_1A_ receptor antagonist, but not by CB_1_R or CB_2_R antagonists (Rodrigues da Silva et al. 2020). A recent clinical trial in patients with schizophrenia found that a 5-HT_1A_ receptor agonist, SEP-363856, was more effective than placebo at reducing psychosis symptom scores after 4 weeks of treatment, highlighting the potential relevance of serotonergic pathways to the treatment of psychosis (Koblan et al. 2020).

A single in vitro study reported that CBD may act as a partial agonist at dopamine D_2_ receptors (Seeman 2016). This was the first study to report such an effect and it requires replication. Moreover, if CBD is primarily acting on dopaminergic pathways, it is surprising that it does not cause akathisia, an effect which is observed with all other partial agonists (Frankel and Schwartz 2017).

Finally, an important consideration for clinical trials of CBD is that it is a potent inhibitor of cytochrome P450 (CYP) enzymes (Brown and Winterstein 2019). Treatment with CBD might therefore increase the plasma levels of some antipsychotic medications and thereby alter their effects. This possibility has yet to be examined in clinical trials of CBD in psychosis. It was explored in a previous trial of adjunctive CBD treatment, but the numbers of participants taking different antipsychotics were too small to permit statistical analysis (McGuire et al. 2018). Potential effect of CBD on the metabolism of other drugs has also been an issue in assessing the effectiveness of CBD in epilepsy (Groeneveld and Martin 2020). CYP enzymes are also present in the brain (Ferguson and Tyndale 2011) and may even contribute to endocannabinoid metabolism (Zelasko et al. 2015).

### Evidence for the effectiveness of CBD in CHR subjects

Recent functional neuroimaging studies have found differences in brain activation in the hippocampus, striatum, insula and midbrain between CHR individuals and healthy controls. A single dose (600 mg) of CBD attenuated these neural differences in CHR subjects but did not significantly alter symptom levels or have adverse effects (Bhattacharyya et al. 2018b; Wilson et al. 2019; Davies et al. 2020). One week of CBD (600 mg) treatment in the same CHR sample was not associated with significant effects on the symptomatic or cortisol response to an experimental stressor and had no adverse effects (Appiah-Kusi et al. 2020). The results of 3 weeks of treatment in this sample are currently in preparation, and the preliminary data indicate that this longer period of treatment is associated with significant symptomatic as well as neuroimaging effects (Bhattacharyya et al. 2018a; Bossong et al. 2019).

In addition to the amelioration of attenuated psychotic symptoms, a primary aim of intervention in the CHR phase is to reduce the risk of later progression to a psychotic disorder. Assessing whether CBD can influence the risk of transition to psychosis is likely to require treatment over a relatively long duration, as the period of maximal risk is over the first 2 years following clinical presentation (Fusar-Poli et al. 2020). A further consideration is that because only 20% of CHR individuals will develop psychosis in 2 years, a trial of CBD as a preventive treatment would require a sample large enough to yield a transition subgroup of sufficient size to detect an effect on this outcome (Fusar-Poli et al. 2020). Recruitment of sufficiently large CHR samples requires large-scale multi-centre trials, and two such trials have recently started. If it was shown to be effective, CBD would be an excellent candidate treatment for a preventive intervention, as it has a particularly benign side effect profile (Chesney et al. 2020b), a critical requirement for clinical treatment in CHR subjects (McGorry et al. 2009; Morrison et al. 2019) especially if this is over a long period.

### Evidence for the effectiveness of CBD in psychotic disorders

To date, there have only been three clinical trials of CBD in patients with psychosis (Table 1). Leweke et al. (2012) compared 4 weeks treatment with 800 mg CBD as monotherapy with amisulpride. There were no differences in efficacy, an encouraging result as it suggested that CBD could be as effective as an antipsychotic. The sample size was small (*N* = 42; 21 per arm) and the study lacked a placebo comparison. Although the same research group subsequently compared CBD with placebo in patients with psychosis (Leweke et al. 2014), the results have only been published as a conference abstract. Two trials have evaluated CBD as an adjunctive treatment to antipsychotic medication in psychosis. Boggs et al. (2018) found no differences between adjunctive CBD and placebo on either psychotic symptoms or cognitive performance in *N* = 36 patients (*n* = 18 per arm), after 6 weeks of treatment. The daily CBD dose was 600 mg. The largest study to date, by McGuire and colleagues (McGuire et al. 2018), tested a higher dose of CBD (1000 mg) as an adjunctive treatment for 6 weeks in *N* = 88 patients (*n* = 43 in the CBD arm and *n* = 45 in the placebo arm). Compared to placebo, CBD treatment was associated with improvements in both ratings of psychotic symptom severity and the clinician’s overall impression. Neither of the two adjunctive studies assessed blood antipsychotic levels, so the possibility that effects were related to pharmacokinetic interactions between CBD and antipsychotics cannot be excluded.

**Table 1:** Randomised controlled trials of cannabidiol in psychotic disorders

Study	Inclusion criteria	Design	Comparison	N	Results
Leweke et al. 2012	Schizophrenia or schizophreniform psychosis; acutely psychotic (BPRS ≥ 36); age 18–50	Parallel groups; 4 weeks	CBD 800 mg/day vs. amisulpride 800 mg/day; monotherapy	42	No differences between groups on the BPRS or PANSS (i.e. CBD as effective as amisulpride). Fewer adverse effects with CBD (including weight gain, EPSEs and prolactin increase)
Boggs et al. 2018	Schizophrenia; clinically stable; age 18–65	Parallel groups; 6 weeks	600 mg/day vs. placebo; adjuncts to antipsychotic medication	41	No differences between groups for cognitive outcomes or PANSS (i.e. no additional improvement with CBD). Sedation was more prevalent in the CBD group
McGuire et al. 2018	Schizophrenia, schizoaffective or schizophreniform disorder; PANSS score ≥ 60; age 18–65	Parallel groups; 6 weeks	CBD 1000 mg/day vs. placebo; adjuncts to antipsychotic medication	88	CBD group had fewer positive psychotic symptoms (PANSS-positive: = − 1.4, 95% CI = − 2.5 to − 0.2). They were also more likely to have been rated as improved (CGI-I: − 0.5, 95% CI = − 0.8 to − 0.1) or as not severely unwell (CGI-S: = − 0.3, 95% CI = − 0.5 to 0.0). No significant differences in PANSS-total, -negative or -general scores. Similar adverse events in each group

*BPRS*, Brief Psychiatric Rating Scale; *PANSS*, Positive and Negative Syndrome Scale; *EPSEs*, extrapyramidal side effects; *CGI (-I, -S)*, Clinical Global Impression (-Improvement Scale, -Severity Scale)

Collectively, these results suggest that CBD may have significant effects on psychotic symptoms in patients with psychosis. The negative results from the trial by Boggs et al. might reflect its small sample size (*N* = 36) and the use of a lower dose of CBD (600 mg) than in the other trials (800 mg and 1000 mg). However, none of the trials published to date have involved large samples, and the optimal dose of CBD for psychosis is unclear. Another consideration is that the Boggs et al. trial involved patients in the chronic stage of psychosis, with an average age of 47 years. Similarly, in the study by McGuire et al., the average age of participants was 41 years, and the trial by Leweke et al. recruited patients with a mean age of 30 years. None of these studies required that participants were in the early stages of psychosis, and the patients were significantly older than would be found in a first episode or a CHR population. This is an important point, as the response to treatment with antipsychotic medication is different in the early and the chronic phases of psychosis, and it is possible that the same may apply to treatment with CBD. In particular, the response to CBD may be altered by the effects of chronic illness and its treatment with antipsychotic medication. The impact of these potentially confounding factors can be minimised by studying patients in the early phases of psychosis.

Because all of the trials completed to date have involved modest sample sizes, there is a clear need for larger scale trials. In addition, they have all involved relatively short durations of treatment (4–6 weeks), and it is not known if better results could be obtained if treatment was provided for a longer period. Finally, all three trials have been in patients with chronic psychosis who had already been treated with antipsychotic medications for a number of years. The extent to which CBD is useful in patients with first-episode psychosis has yet to be tested in clinical trials.

Neuroimaging studies in first-episode samples have had encouraging results. O’Neill et al. used functional magnetic resonance imaging to assess brain activity in patients with first-episode psychosis (*n* = 15) and in healthy controls (*n* = 19) (O’Neill et al. 2020). Patients were scanned after administration of a single dose of CBD (600 mg) or placebo, while healthy controls received no drug treatment. Compared to healthy controls, under placebo conditions, patients showed differential prefrontal and medial temporal activation, and these differences were partially normalised after administration of CBD. A study using proton magnetic resonance spectroscopy in the same subjects found that, compared to placebo, administration of CBD was associated with reductions in psychotic symptom severity alongside a corresponding increase in hippocampal glutamate levels (O’Neill et al. 2021).

According to ClinicalTrials.gov, a total of seven randomised controlled trials of CBD in psychotic disorders are currently in progress (Table 2), and most are recruiting patients within the first few years of illness. Five are using CBD as an adjunctive therapy to antipsychotic medication, one is comparing CBD monotherapy with risperidone and one is a three-arm study comparing CBD monotherapy with olanzapine and placebo. The range of CBD doses being used is 300 to 1000 mg. The results of these studies will be informative for the clinical utility of CBD in the early phases of psychosis. As well as establishing efficacy, important questions include whether CBD is effective as a monotherapy and whether, as with antipsychotic medications, the effective dose is lower in the early stages of psychosis. Similarly, these trials may also clarify whether the effectiveness of CBD is affected by concurrent recreational cannabis use.

**Table 2:** Forthcoming randomised controlled trials of cannabidiol in schizophrenia and early psychosis registered at ClinicalTrials.gov

Identifier	Title	Sponsor	Inclusion criteria	Comparison	Target N	Primary outcome measure
NCT02504151	Cannabidiol Treatment in Patients With Early Psychosis	Yale University, USA	Schizophrenia or schizoaffective disorder; age 18–65	CBD 800 mg/day vs. placebo for 4 weeks; crossover with a 2-week washout	72	PANSS; CGI-S
NCT02932605	Endocannabinoid Control of Microglia Activation as a New Therapeutic Target in the Treatment of Schizophrenia	UMC Utrecht, Netherlands	Schizophrenia, schizophreniform disorder, schizoaffective disorder or psychosis NOS; onset < 5 years; age 16–40	CBD 600 mg/day vs. placebo for 4 weeks; adjunct to antipsychotics; parallel groups	32	Prefrontal myo-inositol measured using 1H-MRS; PANSS and CGI as secondary outcome measures
NCT02926859	Enhancing Recovery in Early Schizophrenia	Central Institute of Mental Health, Mannheim, Germany	Schizophrenia; first diagnosis ≤ 7 years; PANSS ≤ 75; age 18–65	CBD 800 mg/day vs. placebo for 26 weeks; adjunct to antipsychotics; parallel groups	180	All cause discontinuation; PANSS and CGI as secondary outcome measures
NCT02088060	A Four-week Clinical Trial Investigating Efficacy and Safety of Cannabidiol as a Treatment for Acutely Ill Schizophrenic Patients	Central Institute of Mental Health, Mannheim, Germany	Schizophrenia; onset < 3 years ago; acutely unwell, PANSS ≥ 75; age 18–65	CBD 800 mg/day + olanzapine placebo vs. olanzapine 15 mg/day + CBD placebo vs. olanzapine placebo + CBD placebo for 4 weeks; parallel groups	150	PANSS
NCT04421456	Trial to Investigate the Safety and Efficacy of GWP42003-P Versus Placebo as Adjunctive Therapy in Participants with Schizophrenia Experiencing Inadequate Response to Ongoing Antipsychotic Treatment	GW Research Ltd, UK	Schizophrenia; clinically stable; PANSS ≥ 60 and < 110; age 18–50	CBD 300 mg/day vs. CBD 1000 mg/day vs. placebo; adjunct to antipsychotics; parallel groups	366	PANSS; CGI
NCT04411225	Effects of Cannabidiol (CBD) Versus Placebo as an Adjunct to Treatment in Early Psychosis	University of California, San Diego, USA	First-episode psychosis (onset < 2 years) or attenuated psychosis syndrome; clinically stable; age 16–30	CBD 1000 mg/day vs. placebo for 8 weeks; adjunct to antipsychotics; parallel groups	120	PANSS; MATRICS Consensus Cognitive Battery; SOPS; CGI
NCT04105231	Cannabidiol for Treatment of Recent-onset Psychosis with Comorbid Cannabis Use	University of Copenhagen, Denmark	Schizophrenia spectrum or cannabis induced psychotic disorder; onset < 5 years; cannabis use ≥ 1 × /week; age 18–64	CBD 600 mg/day vs. risperidone 4 mg/day for 7 weeks	130	Abstinence from cannabis; PANSS-positive subscale

*PANSS*, Positive and Negative Syndrome Scale; *CGI-S*, Clinical Global Impression Severity Scale; *1H-MRS*, proton magnetic resonance spectroscopy; *MATRICS*, Measurement and Treatment Research to Improve Cognition in Schizophrenia; *SOPS*, Scale of Prodromal Symptoms; *BPRS*, Brief Psychiatric Rating Scale

### Importance of tolerability and acceptability of treatments in the early phases of psychosis

Non-adherence to antipsychotic medication is a major issue for patients with psychosis. It is associated with reduced quality of life and an increased risk of relapse (Novick et al. 2010; Hayhurst et al. 2014). This issue is particularly important in the early stages of psychosis, as younger patients are less likely to adhere to medications (Hickling et al. 2018). This may partly be because they are more likely to experience adverse effects, one of most commonly reported reasons for non-adherence (Wade et al. 2017). In a meta-analysis of studies examining medication adherence, the association between adverse effects and non-adherence was more pronounced in younger populations (Edgcomb and Zima 2018). This may be a particular issue for antipsychotic medications as they can cause several unpleasant adverse effects such as weight gain, sedation, sexual dysfunction and extrapyramidal symptoms (Young et al. 2015).

In contrast to antipsychotics, CBD has relatively few adverse effects and is well tolerated. In a meta-analysis of randomised controlled trials, the only adverse effect attributable to CBD was diarrhoea (Chesney et al. 2020b). In each of the three clinical trials mentioned above, there were no significant differences in the incidence of adverse effects between CBD and placebo. In a head-to-head comparison with the antipsychotic amisulpride, CBD was associated with significantly fewer extrapyramidal side effects, less weight gain and did not elevate prolactin levels (Leweke et al. 2012). Similarly, Boggs et al. found no difference between adjunctive CBD and placebo in extrapyramidal side effects (Boggs et al. 2018), and McGuire et al. also reported an absence of differences with placebo for weight gain, effects on liver function tests and levels of inflammatory markers and HDL cholesterol (McGuire et al. 2018). Moreover, across these three trials, only two out of a total of 84 subjects who received CBD dropped out because of adverse effects: one complained of sedation, the other gastrointestinal adverse effects. This benign side effect profile of CBD is particularly valuable in the early stages of the disorder, as negative experiences with treatment can have a long-term effects on future adherence (Lambert et al. 2004). Even if initial treatment with CBD was ineffective, a patient may be more likely to consider subsequent medications if their early experiences were not off-putting.

A further difference between CBD and antipsychotic medications is that CBD is not associated with stigma. In fact, the reverse is often the case. Many clinicians will be familiar with their patients using over-the-counter preparations of CBD (Chesney et al. 2020a). Over-the-counter CBD products are extremely popular in the UK; in 2019, over a million people were regular users (Gibbs et al. 2019). Many CBD product users cite its perceived benefits on anxiety, sleep problems, stress and general health and wellbeing (Moltke and Hindocha 2021), a perception which may positively impact adherence to CBD and therefore symptomatic and functional outcomes.

CBD’s low burden of adverse effects and acceptability to patients are especially relevant to intervention in the CHR state, as these individuals are at high risk for psychosis but do not have the disorder. Moreover, the majority will not subsequently transition to psychosis, so if treatment is administered as a preventive intervention, many individuals will receive medication even though they might never have needed it. There is therefore a consensus that if a pharmacological intervention is used in this group, it must have a benign adverse effect profile and not be associated with stigma, ensuring that the benefits of the treatment outweigh its costs (McGorry et al. 2009; Morrison et al. 2019).

### The potentially confounding effects of recreational cannabis and over-the-counter cannabinoids

A high proportion of both CHR and first-episode psychosis subjects currently use, or have previously used, cannabis recreationally. The proportions vary considerably with the local population, but on average, around half of CHR subjects have ever used cannabis, a quarter continue to use it and about one in six meet the criteria for a cannabis use disorder (Farris et al. 2020). In first-episode samples, the prevalence of cannabis use disorder is 36% (95% CI: 31 to 41%), considerably higher than in chronic illness: 21% (95% CI: 17 to 25%) (Hunt et al. 2018). More recently, synthetic cannabinoids, which have very high affinity for cannabinoid receptors and can trigger severe psychotic reactions (Hobbs et al. 2018), have also become available. In one study of psychiatric inpatients, 11% of those with psychosis reported having ever used a synthetic cannabinoid, though the majority of these patients (83%) had no intention of using them ever again (Welter et al. 2017).

In first-episode patients, persistent cannabis use after illness onset exacerbates psychotic symptoms, increases the risk of later relapse and is associated with a relatively poor long-term outcome (Schoeler et al. 2016). In CHR individuals, meta-analyses indicate that lifetime use of cannabis is not associated with an increased risk of transition to psychosis (Kraan et al. 2016; Oliver et al. 2020) but that meeting diagnostic criteria for a cannabis use disorder is (Kraan et al. 2016). A change in cannabis use in people at high risk may also be a predictor of later psychosis. A prospective study of 83 individuals with a family history of schizophrenia (which excluded cannabis users at baseline) found that new onset cannabis use was associated with an increased incidence of psychosis in the next 3 years (χ^2^ = 6.4, p = 0.011) (Padmanabhan et al. 2017). Another study in *N* = 134 CHR subjects found that the incidence of psychosis was higher in those who had continued to use cannabis compared to those who had discontinued cannabis use (χ^2^ = 4.5, p = 0.034) (Valmaggia et al. 2014).

Recreational cannabis use thus has the potential to confound clinical trials of CBD in the early phases of psychosis. In addition, the putative therapeutic effects of CBD could be modified by interactions with the main intoxicating compound in cannabis, THC, which is a partial agonist at both CB_1_Rs and CB_2_Rs and has similar activity to anandamide (Pertwee 2008). THC is not rapidly broken down, and intoxication will typically persist for 3–4 h after administration. Prolonged cannabis use has been shown to affect the expression of cannabinoid receptors, FAAH and endocannabinoids (for a review, see Jacobson et al. 2019). Cannabis use may thus disrupt normal endocannabinoid signalling and the response to CBD.

A further consideration is that among patients with psychosis who are cannabis users, the administration of CBD could alter their recreational use of cannabis. Experimental studies in healthy volunteers have shown that pre-treatment with CBD can reduce the negative effects of THC on paranoia and cognitive function (Bhattacharyya et al. 2010; Englund et al. 2013).

In addition, several functional magnetic resonance imaging studies found that CBD has opposite effects to THC on activity in a variety of cortical and striatal areas (Bhattacharyya et al. 2010, 2012; Winton-Brown et al. 2011). More recently, a trial of CBD in regular cannabis users who wanted to stop found that it increased abstinence and reduced urinary THC metabolites over 4 weeks (Freeman et al. 2020). These studies raise the possibility that in patients with psychosis who are cannabis users, CBD might reduce the risk of cannabis exacerbating their symptoms and improve the chances of them reducing or stopping their cannabis use. Since cannabis use disorders are considerably more common in first-episode psychosis (Hunt et al. 2018), CBD may be a particular useful agent at this stage of the illness.

In view of the above, it might be argued that cannabis users should be excluded from clinical trials of CBD, particularly those recruiting patients in the early stages of psychosis. However, because cannabis use is so common in this population, this could limit inclusion to an unrepresentative subset of patients. An alternative approach would be to permit the inclusion of recreational users but carefully monitor their exposure to cannabis throughout the trial via serial urine and blood testing, and self-report measures, such as the Timeline Followback (Robinson et al. 2014).

Trial participants could also be using over-the-counter cannabinoid preparations, which may contain CBD and other cannabinoids. However, at present, the doses of CBD in these preparations are much smaller than those used in clinical trials, and it is unclear if they have any psychiatric effects (Chesney et al. 2020a).

### The potential of other cannabinoids and endocannabinoid system modulators

Evidence that CBD may be effective in psychosis raises the possibility that other compounds with a similar mechanism of action might also be useful. This highlights the importance of determining which molecular mechanism underlies the effects of CBD in psychosis, as this would indicate the most promising therapeutic target for novel compounds. One candidate mechanism of action is the reduction of endocannabinoid metabolism through inhibition of the enzyme FAAH. Unfortunately, the development of FAAH inhibitors was hampered by serious adverse events in phase 1 trials of one compound (Mallet et al. 2016). Since then, there have not been any studies with FAAH inhibitors in patients with psychosis, though one has been evaluated in otherwise healthy volunteers with cannabis dependence (D’Souza et al. 2019). Compared to placebo, 4 weeks of treatment reduced cannabis withdrawal symptoms and cannabis use, without serious adverse events.

CB_1_R inverse agonists, such as rimonabant, have been tested in several clinical trials of schizophrenia (Meltzer et al. 2004; Sanofi-Aventis 2009; Boggs et al. 2012). However, they proved to be ineffective and concerns about adverse effects on mood and anxiety led to their abandonment as a novel psychiatric treatment (Christensen et al. 2007). Historically, it was thought that CB_2_Rs had little relevance to psychosis, but recent pre-clinical evidence suggests that they may regulate midbrain dopaminergic neurotransmission and could have beneficial effects on central immune function (Cortez et al. 2020). Nevertheless, compounds that act on CB_2_Rs have not yet been tested in human studies.

Tetrahydrocannabivarin (THCV) is a naturally occurring homologue of THC with a complex pharmacological profile. At low doses, it is a CB_1_R neutral antagonist and CB_2_R partial agonist (McPartland et al. 2015), and it may also have effects via GPR55, 5-HT_1A_ receptors and TRP channels (Morales et al. 2017). THCV has not been tested in clinical trials of psychosis. An experimental study examined whether pre-treatment with THCV could block the effects of THC (Englund et al. 2016), but the results were inconclusive.

## Conclusion

If large-scale clinical trials confirm that CBD is effective in the treatment of psychosis, it may be particularly useful in the early phases of the disorder. At present, there are no effective interventions for people at CHR for psychosis, and there is a consensus that pharmacological treatments in this population must have minimal adverse effects, especially if given for a prolonged period as a preventive intervention (McGorry et al. 2009). In first-episode psychosis, CBD may be especially helpful in patients who do not respond to treatment with antipsychotic medications and in patients who are reluctant to take antipsychotics because of concerns about side effects and stigmatisation. Large-scale trials in CHR and first-episode subjects are now needed to confirm the potential utility of CBD in the early phases of psychosis. Future trials could also investigate the molecular mechanism of action of CBD by assessing participants with neuroimaging and peripheral blood measures.
